# The unfolded protein response has a protective role in yeast models of classic galactosemia

**DOI:** 10.1242/dmm.012641

**Published:** 2013-09-25

**Authors:** Evandro A. De-Souza, Felipe S. A. Pimentel, Caio M. Machado, Larissa S. Martins, Wagner S. da-Silva, Mónica Montero-Lomelí, Claudio A. Masuda

**Affiliations:** 1 Instituto de Bioquímica Médica Leopoldo de Meis, Programa de Biologia Molecular e Biotecnologia, Universidade Federal do Rio de Janeiro, Rio de Janeiro, 21941-590, Brazil.; 2 Instituto de Bioquímica Médica Leopoldo de Meis, Programa de Bioquímica e Biofísica Celular, Universidade Federal do Rio de Janeiro, Rio de Janeiro, 21941-902, Brazil.

**Keywords:** Yeast, Galactosemia, UPR, Lithium, Galactose

## Abstract

Classic galactosemia is a human autosomal recessive disorder caused by mutations in the *GALT* gene (*GAL7* in yeast), which encodes the enzyme galactose-1-phosphate uridyltransferase. Here we show that the unfolded protein response pathway is triggered by galactose in two yeast models of galactosemia: lithium-treated cells and the *gal7*Δ mutant. The synthesis of galactose-1-phosphate is essential to trigger the unfolded protein response under these conditions because the deletion of the galactokinase-encoding gene *GAL1* completely abolishes unfolded protein response activation and galactose toxicity. Impairment of the unfolded protein response in both yeast models makes cells even more sensitive to galactose, unmasking its cytotoxic effect. These results indicate that endoplasmic reticulum stress is induced under galactosemic conditions and underscores the importance of the unfolded protein response pathway to cellular adaptation in these models of classic galactosemia.

## INTRODUCTION

Classic galactosemia is a human autosomal recessive disorder caused by deleterious mutations in the *GALT* gene that encodes the enzyme galactose-1-phosphate uridyltransferase. This disease is usually diagnosed soon after birth due to severe clinical symptoms caused by the toxicity of the galactose and lactose ingested from milk and affects more than 1 in 60,000 newborns worldwide (Fridovich-Keil and Walter, 2008). Symptoms of acute galactose toxicity include jaundice, hepatosplenomegaly, hepatocellular insufficiency, food intolerance, hypoglycemia, renal tubular dysfunction, muscle hypotonia, cataracts and sepsis. These symptoms can lead to death if not treated properly. The major treatment for this disease is based on a galactose/lactose-restricted diet, but even well-treated patients can develop other symptoms such as mental retardation, verbal dyspraxia, motor abnormalities and hypergonadotropic hypogonadism (Bosch, 2006; Fridovich-Keil and Walter, 2008).

**Fig. 1.**
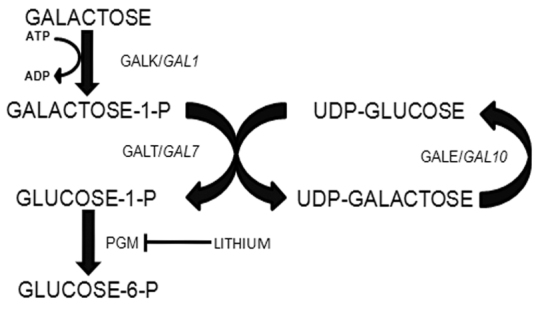
**Schematic representation of the galactose metabolic pathway in yeast and humans.** *GALK* and *GAL1* encode galactokinase in humans and yeast, respectively. *GALT*/*GAL7* and *GALE*/*GAL10* encode the galactose-1-phosphate uridyltransferase and the UDP-galactose 4 epimerase, respectively. PGM indicates the enzyme phosphoglucomutase. The arrows indicate the flux in the direction of catabolism.

Human and yeast cells metabolize galactose via the Leloir pathway (Fig. 1). This pathway includes three enzymes: galactokinase (encoded by the *GALT* gene in humans/*GAL1* in yeast), which phosphorylates galactose using ATP as the phosphate donor, generating galactose-1-phosphate and ADP; galactose-1-phosphate uridyltransferase (*GALT*/*GAL7*), which transfers the uridylmonophosphate group from an UDP-glucose molecule to the galactose-1-phosphate, generating UDP-galactose and glucose-1-phosphate; and UDP-galactose 4-epimerase (*GALE*/*GAL10*), which catalyzes the isomerization of UDP-galactose to UDP-glucose. The glucose-1-phosphate generated by the Leloir pathway can then be converted to glucose-6-phosphate by phosphoglucomutases (*PGM*/*PGM1-3*) and be utilized by a series of metabolic pathways such as glycolysis and the pentose-phosphate pathway, among others (Kosterlitz, 1943; Leloir, 1951; Kalckar, 1957).

The unfolded protein response (UPR) is mainly a cell protective response triggered when unfolded proteins accumulate in the lumen of the endoplasmic reticulum (ER) during conditions of ER stress (Walter and Ron, 2011). Recently, it has been shown that signals different from unfolded proteins can also induce UPR activation (Kimata and Kohno, 2011). There is evidence in the literature suggesting that the UPR is activated in galactosemia. In a human cell line model of classic galactosemia, Slepak and co-workers showed that genes controlled by the UPR (e.g. *BIP*, *ERO1L*, *CHOP*, *ATF4*, *XBP1*, *GADD45A*, *ATF3*) are induced by galactose treatment (Slepak et al., 2007). This same group showed by microarray that in a yeast model of classic galactosemia, the *gal7*Δ mutant, genes controlled by the UPR (e.g. *ERO1*, *KAR2*) are also induced by galactose (Slepak et al., 2005). Our group has been studying the effect of lithium in yeast cells and has described that, in the presence of this ion, galactose induces a cellular stress that resembles that observed in classic galactosemia because it leads to the accumulation of galactose-1-phosphate due to the inhibition of phosphoglucomutase (Masuda et al., 2001; Masuda et al., 2008). We have also observed in microarray experiments that the *ERO1* gene is also induced under this condition (Bro et al., 2003). Recently, Nagy and co-workers showed that lithium induced UPR in galactose-grown Jurkat cells (Nagy et al., 2013). Despite all of this evidence of UPR activation in classic galactosemia models, none of these works carefully characterized this response nor did they probe the importance of UPR activation under these circumstances.

In this work, we explored two yeast models of classic galactosemia (lithium-treated cells and the *gal7*Δ mutant) to show that the UPR is activated by galactose and has a protective role against the cytotoxic effect of galactose under these conditions. We also present evidence that UPR activation is dependent on galactokinase activity. These results suggest that galactose-1-phosphate synthesis, and possibly its accumulation, is essential to cause the ER stress that triggers UPR activation under galactosemic conditions.

**TRANSLATIONAL IMPACT**

**Clinical issue**

Classic galactosemia is a hereditary disease affecting 1:60,000 newborns and is caused by mutations in the *GALT* gene that encodes the enzyme galactose-1-phosphate uridyltransferase. Disease onset, which occurs soon after birth in response to galactose exposure via milk ingestion, is characterized by a series of acute symptoms such as jaundice, food intolerance, hypoglycemia and sepsis. If not treated properly, these symptoms can lead to death. However, even when correctly treated with a galactose-restricted diet, a considerable number of patients develop long-term symptoms such as premature ovarian failure, verbal and cognitive developmental problems and motor abnormalities. Galactose-1-phosphate accumulation is a hallmark of classic galactosemia and seems to be an important factor contributing to the symptoms of this disease; however, the molecular mechanisms of toxicity are poorly understood.

**Results**

Earlier work suggested that the unfolded protein response (UPR), a protective response that is important for cellular adaptation under conditions of endoplasmic reticulum stress, is activated in galactosemia. In this study, the authors use two previously established yeast models of classic galactosemia – the *gal7*Δ strain and lithium-treated cells – to explore this phenomenon. They demonstrate that the UPR is activated in a galactose-dependent manner. Furthermore, they show that galactose-1-phosphate synthesis is essential for galactose-induced UPR, and also provide evidence that the activation of the stress response protects yeast cells against the cytotoxic effects of galactose in the two models.

**Implications and future directions**

These findings indicate that, under galactosemic conditions, endoplasmic reticulum stress triggers the activation of the UPR, underscoring the importance of this response in protecting the cell against cytotoxicity caused by galactose-1-phosphate or a derived metabolite. These results support the development of galactokinase inhibitors as drugs for the treatment of classic galactosemia. In addition, the work suggests that molecules that interfere with endoplasmic reticulum stress might be viable drug candidates to treat this disease. Elucidation of the complete role of the UPR in supporting cell survival in galactosemic patients awaits investigation in additional models of the disease.

## RESULTS

### Galactose-dependent activation of UPR in yeast models of galactosemia

**Fig. 2.**
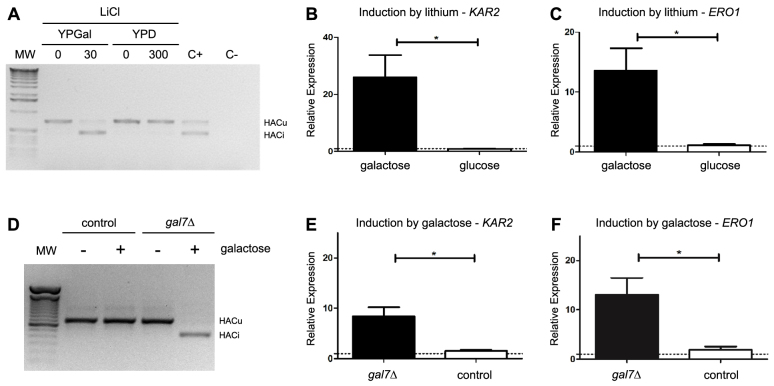
**The UPR is triggered in the presence of galactose in both models of galactosemia.** (A) The splicing of the *HAC1* mRNA was followed by RT-PCR in control cells grown in medium containing either glucose (YPD) or galactose (YPGal) as the main carbon source, before (0) or after a 2-hour incubation with LiCl (30 or 300 mM). We treated cells growing in YPD with 300 mM LiCl instead of 30 mM used in YPGal because we could not detect any decrease in growth at lower concentrations when glucose was the main carbon source. C− indicates a PCR reaction without the addition of cDNA; C+ indicates a sample of yeast cells treated for 1 hour with 2.5 μg/ml of tunicamycin, a known inducer of UPR. MW, molecular weight marker. HACu indicates the product of the unspliced mRNA and HACi indicates the product of the spliced mRNA. A representative result of three independent experiments is shown. (B,C) Relative expression levels of *KAR2* and *ERO1* were determined by qRT-PCR from control cells grown in YPD or YPGal, in the presence or absence of lithium. Induction by lithium was calculated using the comparative Ct method and compared with the expression before and after the 2-hour incubation with lithium (300 mM in YPD and 30 mM in YPGal). Results are the mean ± s.d. of four independent experiments. (D) Splicing of the *HAC1* mRNA was followed by RT-PCR from control or *gal7*Δ cells grown in YPGly medium and treated (+) or not (−) with 0.02% galactose for 2 hours. A representative result of three independent experiments is shown. (E,F) Relative expression levels of *KAR2* and *ERO1* were determined by qRT-PCR from control or *gal7*Δ cells grown in YPGly medium and treated or not with 0.02% galactose. Induction by galactose was calculated by comparing the expression before and after the 2-hour incubation period with galactose. Results are the mean ± s.d. of four independent experiments. *P<0.05, Student’s *t*-test.

In yeast, the ER transmembrane protein Ire1p is the sensor of unfolded proteins in the lumen of the ER. Once Ire1p is activated, it catalyzes the splicing of the *HAC1* mRNA via its intrinsic RNase activity together with the RNA ligase activity of Trl1p (Walter and Ron, 2011). Because the splicing of the *HAC1* mRNA is an essential event during UPR activation in yeast, we followed this event by RT-PCR experiments as an indicator of UPR activation in both models of galactosemia. Fig. 2A shows that *HAC1* mRNA is spliced when wild-type yeast cells are exposed to lithium in the presence of galactose, but not in the presence of glucose. Galactose also induced the splicing of *HAC1* mRNA in the *gal7*Δ strain, but not in the control strain (Fig. 2D).

Hac1p is a transcription factor that activates specific genes when the UPR is active (Walter and Ron, 2011). Another way to follow UPR activation is to monitor the relative expression of Hac1p target genes such as *KAR2* and *ERO1* (Cox et al., 1993; Takemori et al., 2006), which encode an ER resident chaperone and a thiol oxidase, respectively. Lithium induced the expression of both *KAR2* and *ERO1* (Fig. 2B,C) in the presence galactose, but not in the presence of glucose. Galactose also induced the expression of both genes in the *gal7*Δ mutant, but not in the control strain (Fig. 2E,F). These results indicate that the UPR is active under ‘galactosemic’ conditions in both yeast models.

### Galactose-1-phosphate synthesis is essential for galactose-induced UPR activation in yeast models of galactosemia

**Fig. 3.**
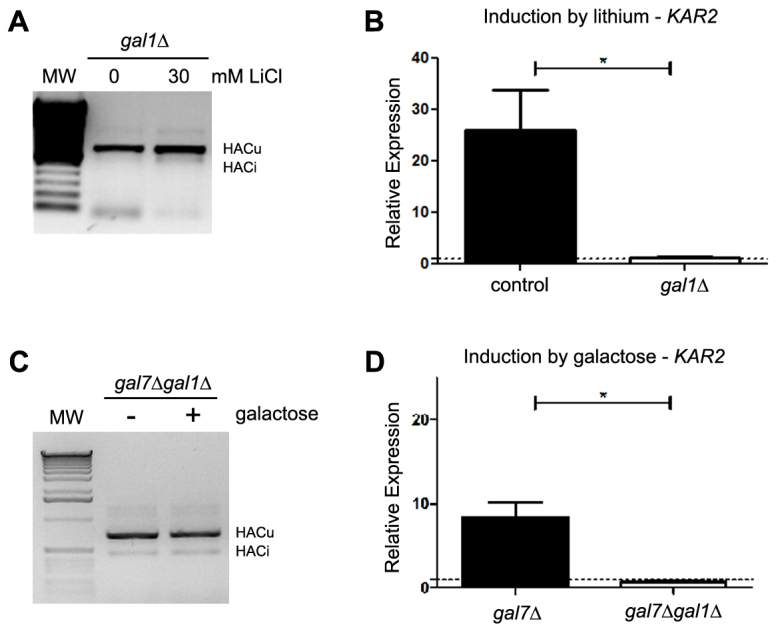
**Galactose-induced UPR activation depends on galactokinase.** (A) The splicing of *HAC1* mRNA was assessed by RT-PCR from strain *gal1*Δ grown in YPGal before and after a 2-hour treatment with 30 mM LiCl. MW, molecular weight marker. HACu indicates the product of the unspliced mRNA and HACi indicates the product of the spliced mRNA. A representative result of three independent experiments is shown. (B) Relative expression of the *KAR2* gene was determined by qRT-PCR in control and *gal1*Δ cells grown in YPGal before and after a 2-hour treatment with 30 mM LiCl. Results are the mean ± s.d. of three independent experiments. (C,D) Similar experiments were performed using *gal7*Δ and *gal7*Δ*gal1*Δ strains grown in YPGly medium and treated or not for 2 hours with 0.02% galactose. *P<0.05, Student’s *t*-test.

Galactose-1-phosphate accumulation is a hallmark of classic galactosemia. There is a body of experimental data that points to a toxic role for galactose-1-phosphate accumulation, although the molecular mechanism behind this toxicity is poorly understood (Lai et al., 2009). However, there is also evidence that other molecules could contribute to the symptoms of galactosemia (Mumma et al., 2008; Lai et al., 2009). To test whether the galactose-dependent UPR activation observed is dependent on the synthesis of galactose-1-phosphate, we deleted the galactokinase-encoding gene *GAL1* in both models and assessed UPR activation. The deletion of the galactokinase gene blocked the galactose-dependent UPR activation in both the lithium-induced (Fig. 3A,B) and the *gal7*Δ mutant (Fig. 3C,D) models of galactosemia. This conclusion is supported by both UPR activation assays: the splicing of *HAC1* mRNA (Fig. 3A,C) and expression of the UPR target genes *KAR2* (Fig. 3B,D) and *ERO1* (data not shown). It is worth noting that galactokinase deletion protects yeast cells from the toxic effect of galactose in both models (supplementary material Fig. S1A,B) (Douglas and Hawthorne, 1964; Ross et al., 2004; Masuda et al., 2008), but did not increase tolerance to other ER stressors such as tunicamycin (supplementary material Fig. S1C) or dithiothreitol (DTT), indicating that this protection is not due to an increase in tolerance to ER stress in general. Independently of whether galactose-1-phosphate directly or indirectly induces UPR, these results indicate that galactose-1-phosphate synthesis is essential for the promotion of the ER stress by galactose that triggers the UPR.

### The UPR has a protective role against galactose cytotoxicity in yeast models of galactosemia

**Fig. 4.**
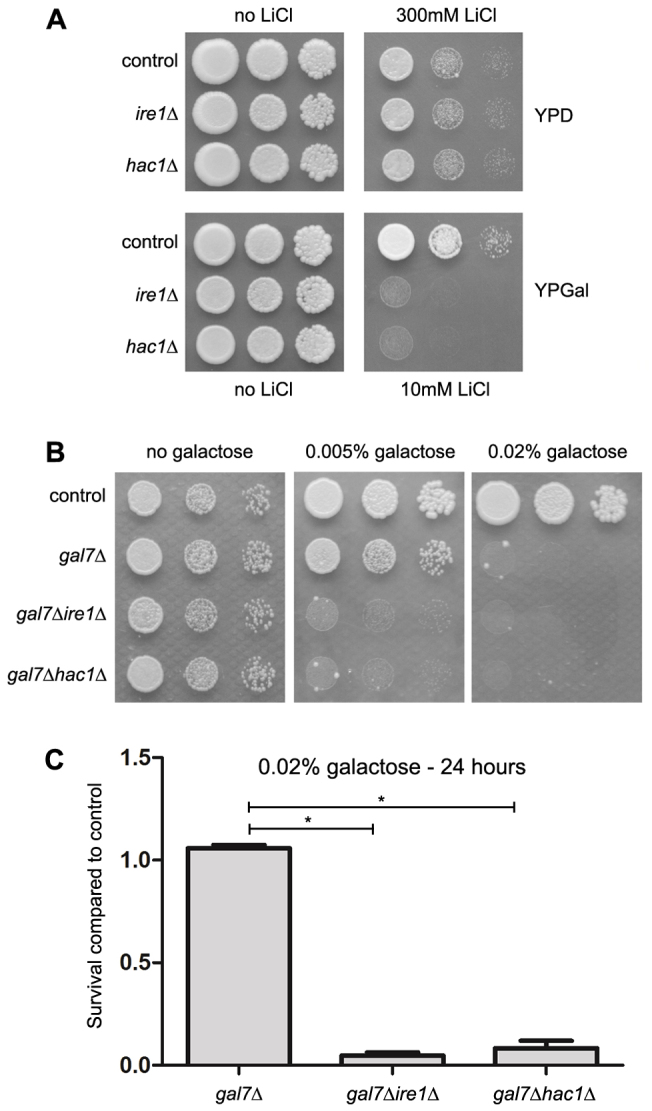
**The UPR is important for cell adaption and survival under galactosemic conditions.** (A) The indicated yeast strains were grown in YPGal medium until the stationary phase and diluted to O.D._600nm_ values of 0.3, 0.03 and 0.003 in sterile water. Approximately 5 μl of each cell suspension was plated in the indicated medium and incubated for 2 (YPD) or 3 (YPGal) days at 30°C. A representative result of three independent experiments is shown. (B) The indicated strains were grown in YPGly medium for 48 hours, plated as described above in the indicated medium and incubated for 4 days at 30°C. A representative result of three independent experiments is shown. (C) The indicated yeast strains were grown in duplicate in YPGly medium until early-log phase (O.D._600nm_ of 0.1). At this point, galactose was added to a final concentration of 0.02% in one of the cultures. Aliquots of the cultures were taken 24 hours after the addition of galactose. Cell suspensions were normalized by O.D._600nm_ and ~200 cells were inoculated per YPD plate. Colony-forming units (cfu) were counted after 2 days at 30°C, and the survival rate of mutant strains was compared with the results of the control strain. Results are the mean ± s.d. of three independent experiments. *P<0.05, Student’s *t*-test.

The UPR is a cellular response that has a protective role when yeast cells are under endoplasmic reticulum stress (Walter and Ron, 2011). To test whether UPR has a protective role under ‘galactosemic’ conditions, we first compared the lithium tolerance of yeast cells that are unable to activate UPR (*ire1*Δ and *hac1*Δ) with the tolerance of a control yeast strain. Fig. 4A shows that UPR activation is important for lithium tolerance when cells are grown in medium containing galactose (YPGal), but not when the main carbon source is glucose (YPD). We also observed that UPR-negative *gal7*Δ strains (*gal7*Δ*ire1*Δ and *gal7*Δ*hac1*Δ) were even more sensitive to galactose than the UPR-positive *gal7*Δ strain (Fig. 4B). It is important to note that the *ire1*Δ strain did not accumulate significantly more galactose-1-phosphate than its control strain when treated with lithium and galactose for 2 hours (control versus *ire1*Δ: 19.36±3.02 versus 25.08±2.27 nmol/mg dry weight, *P*=0.20). Similarly, *gal7*Δ*ire1*Δ did not accumulate more galactose-1-phosphate than the *gal7*Δ strain (*gal7*Δ versus *gal7*Δ*ire1*Δ: 49.40±1.80 versus 36.02±5.99 nmol/mg dry weight, *P*<0.05). Deletion of *IRE1* or *HAC1* did not increase the sensitivity to other stressful conditions not directly related to ER stress such as growth at 37°C, growth in the presence of high concentrations of NaCl, sorbitol, sodium arsenite or hydrogen peroxide (Gardarin et al., 2010) (supplementary material Fig. S2 and data not shown). These results indicate that the increased sensitivities to lithium and galactose induced by the disruption of UPR in these models are not caused by an increase in galactose-1-phosphate accumulation nor due to a general, nonspecific sickness of these strains. It has been previously shown that galactose incubation for up to 48 hours has a cytostatic, but not cytotoxic, effect on the *gal7*Δ yeast strain (Ross et al., 2004; Slepak et al., 2007). Because the assays presented in Fig. 4A,B could not discern whether the increased sensitivity to galactose is due to an increased cytostatic or cytotoxic effect, we tested the effect of galactose on the UPR-positive and UPR-negative *gal7*Δ strains using a cell viability assay. Fig. 4C shows that 0.02% galactose was cytotoxic to *gal7*Δ UPR-negative cells but not to *gal7*Δ UPR-positive cells. Similar results were obtained with the lithium-treatment model (supplementary material Fig. S3). Together, these results indicate that ‘galactosemic’ conditions promote ER stress that triggers the activation of the UPR and underscores the importance of the UPR in the protection against the cytotoxicity caused by galactose in these classic galactosemia models.

## DISCUSSION

The results presented here support the idea that molecules that interfere with ER stress (Kraskiewicz and FitzGerald, 2012) might be good drug candidates to treat classic galactosemia. Chemical chaperones are one class of these molecules that help stabilize the folded structure of proteins. In a rat model of galactosemia, chemical chaperones could partially suppress UPR activation in lens epithelial cells and partially protect rats against cataract formation induced by a 50% galactose chow diet (Mulhern et al., 2007). However, because normal rat strains with an intact Leloir pathway were used in that model, it is somewhat difficult to compare their results with ours. Furthermore, the same group showed that in cultured lens epithelial cell lines, the UPR can be induced by treatment with high concentrations (100–125 mM) of galactose, glucose, mannose, mannitol or NaCl. These results suggest that, in this model, the UPR is likely activated by osmotic stress and not by the accumulation of any specific metabolite such as galactose-1-phosphate (Mulhern et al., 2006). Recently, others have also proposed the use of chemical chaperones as drug candidates for classic galactosemia based on the protein structural instability caused by disease-associated mutations on the human *GALT* gene (McCorvie et al., 2013).

We have tested the effect of the chemical chaperone 4-phenylbutyric acid (4-PBA) on both yeast models of galactosemia used in this work (supplementary material Fig. S4) but we did not observe any protective effect of 4-PBA in growth assays (supplementary material Fig. S4B,C). One possible explanation for these negative results is that yeast seems to be less susceptible to these agents than mammalian cells. For instance, as far as we know, the only experiments showing some protective effect on growth against the ER stressor and UPR inducer tunicamycin were performed in the UPR-negative strain *ire1*Δ (Kubota et al., 2006) (supplementary material Fig. S4A). On the other hand, it has been proposed that UPR could be activated by signals other than the accumulation of unfolded protein in the ER, such as small molecules (flavonoids) and membrane alterations (Kimata and Kohno, 2011). The lack of effect of 4-PBA, even in UPR-negative strains (supplementary material Fig. S4B,C), suggests that one of those other signals could be responsible for UPR activation under galactosemic condition. Further studies are necessary to better understand at the molecular level the UPR activation process under these conditions.

We have provided clear evidence that galactose-1-phosphate synthesis is essential to the induction of ER stress and, consequently, to UPR activation in these models (Fig. 3). However, the molecular mechanism behind this process is still unknown. One hypothesis is that ER stress is caused by defects in protein glycosylation. Several studies have reported that erroneous protein glycosylation occurs in patients and cell models of classic galactosemia (Dobbie et al., 1990; Ornstein et al., 1992; Charlwood et al., 1998; Lai et al., 2003). Lai and co-workers proposed a mechanism in which galactose-1-phosphate inhibits the enzyme UDP-glucose pyrophosphorylase and decreases the pool of both UDP-glucose and UDP-galactose, thereby interfering with glycosylation reactions (Lai et al., 2003).

Another hypothesis to explain how galactose can cause ER stress in galactosemia would be a defect in inositol metabolism and, consequently, in calcium homeostasis. It is known that galactose-1-phosphate can function as an alternative substrate for inositol monophosphatases (Parthasarathy et al., 1997), and it was proposed that the accumulation of galactose-1-phosphate can cause a decrease in the levels of free inositol as a result of competition (Bhat, 2003; Slepak et al., 2007). This reduction would lead to changes in the phosphatidylinositol levels, interfering with calcium homeostasis in the ER. Slepak and co-workers showed a defect in calcium homeostasis in a human cell line model of classic galactosemia (Slepak et al., 2007).

Although we cannot rule out a role for the inhibition of inositol monophosphatases in causing the ER stress, the results obtained in this work with lithium indicate that the inhibition of inositol monophosphatases might not be sufficient to cause it. Lithium is also an inhibitor of inositol mono- and polyphosphatases (Hallcher and Sherman, 1980; Gee et al., 1988; Inhorn and Majerus, 1988) and is believed to cause inositol depletion in cells (Berridge et al., 1989), including in yeast (Vaden et al., 2001), by a similar mechanism as proposed for galactose-1-phosphate. However, although lithium could induce the UPR in control yeast strains growing in galactose, it could not induce the UPR in yeast growing in glucose (Fig. 2A,B) or in the *gal1*Δ mutant growing in galactose (Fig. 3A,B). Inositol supplementation to the medium also did not confer tolerance to galactose in the *gal7*Δ model (data not shown). Furthermore, the yeast strain that had deletions of both inositol monophosphatase genes, *inm1*Δ*inm2*Δ, grew normally on YPGal medium (Masuda et al., 2008). These results indicate that the inhibition of inositol monophosphatase is not sufficient to cause ER stress and activate the UPR in yeast.

Another possibility is that the UPR activation and toxicity are caused by an energy starved condition due to interruption of the galactose utilization pathway in a similar way to how glucose starvation induces UPR and toxicity (Pouysségur et al., 1997; Schröder and Kaufmann, 2005; Sun et al., 2011). However, in our experimental conditions we use rich media with carbon sources other than galactose, such as peptone and glycerol. Furthermore, if the interruption of the Leloir pathway were the main problem, it would not be expected that the introduction of another blockage in the pathway with the *GAL1* deletion could suppress the effect of the downstream blockage with lithium or *GAL7* deletion.

The hypothesis that galactose-1-phosphate accumulation plays a role in galactose-induced symptoms and toxicity in different organisms is supported by a number of experimental results. Nevertheless, there is also strong evidence that galactose-1-phosphate accumulation is not the sole culprit of galactose toxicity under galactosemic conditions. For example, modifications in the UDP-glucose and/or UDP-galactose levels seem to be important determinants of phenotypes under these conditions (Mumma et al., 2008; Lai et al., 2009). Based on our results, we cannot be certain that UPR is induced due to the galactose-1-phosphate accumulation observed in our models. However, independently of the molecular mechanism, the observation that *GAL1* deletion completely inhibited the galactose-dependent UPR activation (Fig. 3) and galactose toxicity in these models (supplementary material Fig. S1) (Douglas and Hawthorne, 1964; Ross et al., 2004; Masuda et al., 2008) represents further evidence that the inhibition of galactokinase is a good strategy to treat classic galactosemia. Currently, there is a promising ongoing effort toward this goal that uses a combination of computational and biochemical high-throughput screenings to identify and design small molecule inhibitors targeting the human galactokinase (Tang et al., 2012).

The main role of the UPR is to protect cells from the deleterious effects of unfolded protein accumulation in the lumen of the endoplasmic reticulum (Walter and Ron, 2011). In order to perform this role, cells first have to sense unfolded proteins or other signals in the ER and transduce this signal to the rest of the cell so that a proper response (mainly a transcriptional response) can be arranged. In yeast, Ire1p is the only UPR sensor. In metazoan cells, however, there are three distinct UPR sensors in the secretory pathway that give rise to different cellular responses: an Ire1 homolog, PERK and ATF6. Although short-term UPR activation also induces a mainly protective UPR response in metazoans, if this activation is prolonged enough the UPR can trigger pro-apoptotic pathways (Walter and Ron, 2011; Kraskiewicz and FitzGerald, 2012). Therefore, if the UPR is induced in the cells of galactosemic patients (Slepak et al., 2007), it is possible that the UPR has a dual antagonistic effect on cell survival – an initial protective role in acute, short-term ER stress and a pro-apoptotic role after a prolonged ER stress – as has been shown in other diseases such as retinitis pigmentosa, atherosclerosis and type II diabetes (Engin and Hotamisligil, 2010; Walter and Ron, 2011). The importance of these two aspects of the UPR in the pathophysiology of classic galactosemia needs to be addressed in more suitable models.

## MATERIALS AND METHODS

### Yeast strains and media

*Saccharomyces cerevisiae* wild-type strain BY4741 (MATa, *his3*Δ*1*, *leu2*Δ*0*, *met15*Δ*0*, *ura3*Δ*0*) and *lys2Δ* were used as the control strains. We used the *lys2*Δ strain as a second control strain because during the course of the work, we observed that most strains coming from the MATa deletion library (Open Biosystems, USA) were slightly more tolerant to lithium and galactose than BY4741 (supplementary material Fig. S5). There is no evidence in the literature that deletion of any of the genes tested in supplementary material Fig. S5 is related to galactose metabolism. All figures show the results using *lys2*Δ as control. Strains deleted for *gal1*Δ, *gal7*Δ, *hac1*Δ and *ire1*Δ were obtained from the MATa deletion library. The double disrupted strains *gal7*Δ*gal1*Δ, *gal7*Δ*hac1*Δ and *gal7*Δ*ire1*Δ were constructed by one-step gene replacement by inserting the HIS3MX6 marker in the place of *GAL1*, *HAC1* or *IRE1* in the *gal7*Δ strain (Brachmann et al., 1998). All yeast cells were transformed by the lithium acetate method (Gietz and Schiestl, 2007). The following primers for the construction of deletion cassettes were used: GAL1KAN5 (5′-TAATATACCTCTATACTTTAACGTCAAGGAGAAAAAACTATAATGCGTACGCTGCAGGTCGAC-3′), GAL1KAN3 (5′-AATGAGAAGTTGTTCTGAACAAAGTAAAAAAAAGAAGTATACTTATCGATGAATTCGAGCTCG-3′), HAC1KAN5 (5′-TAACAACCTCCTCCTCCCCCACCTACGACAACAACCGCCACTATGCGTACGCTGCAGGTCGAC-3′), HAC1KAN3 (5′-TGTCAAGATCAATTGAATTGTCAAAGGGTAGACTGTTTCCCGCTATCGATGAATTCGAGCTCG-3′), IRE1KAN5 (5′-AAACAGCATATCTGAGGAATTAATATTTTAGCACTTTGAAAAATGCGTACGCTGCAGGTCGAC-3′), IRE1KAN3 (5′-ATGCAATAATCAACCAAGAAGAAGCAGAGGGGCATGAACATGTTATCGATGAATTCGAGCTCG-3′).

The deletion of the respective genes was confirmed by PCR using the following primers: GAL1A (5′-ACGAATCAAATTAACAACCATAGGA-3′), GAL1B (5′-AAGTAATTAGACCAGTCCGACACAG-3′), GAL1C (5′-AAACTTTACGAATGTTCTTGTCCAG-3′), GAL1D (5′-ATGTCAAGAATAGGTATCCAAAACG-3′), HAC1A (5′-ATACATTTATGAGGGTTGTAAGGCA-3′), HAC1B (5′-GCAGCTCTTCTGTTTCTCAAAATAC-3′), HAC1C (5′-GTATTTTGAGAAACAGAAGAGCTGC-3′), HAC1D (5′-GAAAAGAATGGCTCTATTTGTTCAG-3′), IRE1A (5′-AATAGGTTTTCGCTATTTTATTGCC-3′), IRE1B (5′-CACCATAGTTGAAATTGAGCTTTTT-3′), IRE1C (5′-CCGTTAAAAAGACCTACTGCTATGA-3′), IRE1D (5′-TCACAAAGATTAAAGGAGCTATTGG-3′), kanB (5′-CTGCAGCGAGGAGCCGTAAT-3′) and kanC3 (5′-CCTCGACATCATCTGCCCAGAT-3′).

Yeast cells were grown at 30°C in YP medium (1% yeast extract, 2% Bacto peptone) containing 2% glucose (YPD), 2% galactose (YPGal) or 2% glycerol (YPGly). Lithium chloride and galactose (Sigma-Aldrich, USA) were added to the medium as reported.

### *HAC1* transcript splicing analysis

Total RNA was extracted as previously described (Schmitt et al., 1990) from cells grown to the exponential phase (~0.8 O.D._600nm_) under the conditions described in each experiment. A total of 1 μg of total RNA was used to prepare the first-strand cDNA using the High Capacity cDNA Reverse Transcription Kit following the manufacturer’s protocol (Applied Biosystems, Foster City, CA). The cDNA was used as a template for the amplification of *HAC1* cDNA by PCR. The PCR conditions were 94°C for 1 minute followed by 34 cycles at 94°C for 45 seconds, 54°C for 45 seconds, 72°C for 60 seconds and finally 72°C for 7 minutes. The primers used in these reactions were *HAC1F* (5′-CTGGCTGACCACGAAGACGC-3′) and *HAC1R* (5′-TTGTCTTCATGAAGTGATGA-3′). The PCR product includes the intron that is removed when UPR is activated, which enabled us to detect a 720-bp band when the *HAC1* transcript was not spliced and a 470-bp band when the *HAC1* transcript was spliced (Mori et al., 2010).

### Quantification of the *ERO1* and *KAR2* transcripts by qRT-PCR

Total RNA extraction and first-strand cDNA synthesis were performed as described above. cDNA preparations were used as the template for amplification using the Power SYBR-Green PCR master mix (Applied Biosystems, USA). The PCRs were performed using either the ABI 7500 or the StepOnePlus Real-Time PCR systems (Applied Biosystems). The PCR conditions were as follows: 95°C for 10 minutes; 40 cycles at 95°C for 15 seconds and 60°C for 1 minute; the melting curve was 95°C for 15 seconds, 60°C for 1 minute and 95°C for 15 seconds. The relative expression levels were calculated using the comparative Ct method (Livak and Schmittgen, 2001) using *ACT1* and/or *TFC1* as reference genes (Teste et al., 2009). The number of PCR cycles required to reach a fluorescence intensity greater than the set threshold (Ct) was calculated using Sequence Detection Software, version 1.3 (Applied Biosystems). The following primer pairs were used: *ACT1F* (5′-TTCCCAGGTATTGCCGAAA-3′), *ACT1R* (5′-TTGTGGTGAACGATAGATGGA-3′), *ERO1F* (5′-AACGCCGTTCTGATTGATTT-3′), *ERO1R* (5′-GATTCACCAGTTTCGCCAAT-3′), *KAR2F* (5′-TGACAACCAACCAACCGTTA-3′), *KAR2R* (5′-TACACCTCTTGGTGCTGGTG-3′), *TFC1F* (5′-TGGATGACGTTGATGCAGAT-3′) and *TFC1R* (5′-GCTCGCTTTTCATTGTTTCC-3′).

### Plate growth assay

Cells were grown in YPD, YPGal or YPGly medium to stationary phase, and serial dilutions of the cultures were prepared in sterile-distilled water to O.D._600nm_ values of 0.3, 0.03 and 0.003. Approximately 5 μl of each dilution was spotted onto the medium described in the experiments. Plates were incubated at 30°C for 2–5 days and then photographed using a Canon 20D camera. Images were processed using Adobe Photoshop software.

### Cell viability assay

The yeast strains *gal7*Δ, *gal7*Δ*ire1*Δ, *gal7*Δ*hac1*Δ and *lys2*Δ (control strain) were grown overnight in YPD and diluted to 0.02 O.D._600nm_ in YPGly medium. After a 16-hour incubation period at 30°C with agitation, galactose was added to YPGly to a final concentration of 0.02%. After 24 hours of treatment, ~200 cells were plated on YPD agar plate and incubated at 30°C for 2 days. The number of colonies was counted, and the survival rate of mutants was calculated by comparing the cells incubated with galactose to the control condition.

### Extraction and analysis of galactose-1-phosphate content

Yeast cells were inoculated in YPGal (control and *ire1*Δ) or YPGly (*gal7*Δ and *gal7*Δ*ire1*Δ) media and incubated for 6 hours at 30°C with agitation until O.D._600nm_ ~0.8 was reached. At this point, LiCl (30 mM) or galactose (0.02%) was added to YPGal or YPGly media, respectively, and the cultures were returned to the incubator. After 2 hours, ~5×10^8^ cells were collected by rapid vacuum filtration in 0.45-μm Millipore filters, washed quickly with 10 ml of H_2_O and heated at 80°C for 3 minutes in 3 ml of a 75% ethanol solution. Samples were lyophilized, re-suspended in H_2_O at an equivalent volume to 2×10^9^ cells/ml and centrifuged at 16.000 rpm for 10 minutes at 4°C. Aliquots of 10 μl were used in the analysis of the galactose-1-phosphate content as described previously (Masuda et al., 2008).

## Supplementary Material

Supplementary Material
