# Supplementary material for: The unfolded protein response has a protective role in yeast models of classic galactosemia
Source: Dis Model Mech. 2013 Sep 25;7(1):55–61. doi: 10.1242/dmm.012641 (PMC3882048; doi:10.1242/dmm.012641)
Supplement: Supplementary Material [file supp_7_1_55__index.html]

The unfolded protein response has a protective role in yeast models of classic galactosemia — The unfolded protein response has a protective role in yeast models of classic galactosemia — Supplementary Material 

# The unfolded protein response has a protective role in yeast models of classic galactosemia

## DMM012641 Supplementary Material

**Files in this Data Supplement:**

- **Supplementary Material PDF**
