# Supplementary material for: The unfolded protein response has a protective role in yeast models of classic galactosemia
Source: Dis Model Mech. 2013 Sep 25;7(1):55–61. doi: 10.1242/dmm.012641 (PMC3882048; doi:10.1242/dmm.012641)
Supplement: Supplementary Material [file supp_012641_DMM012641.pdf]

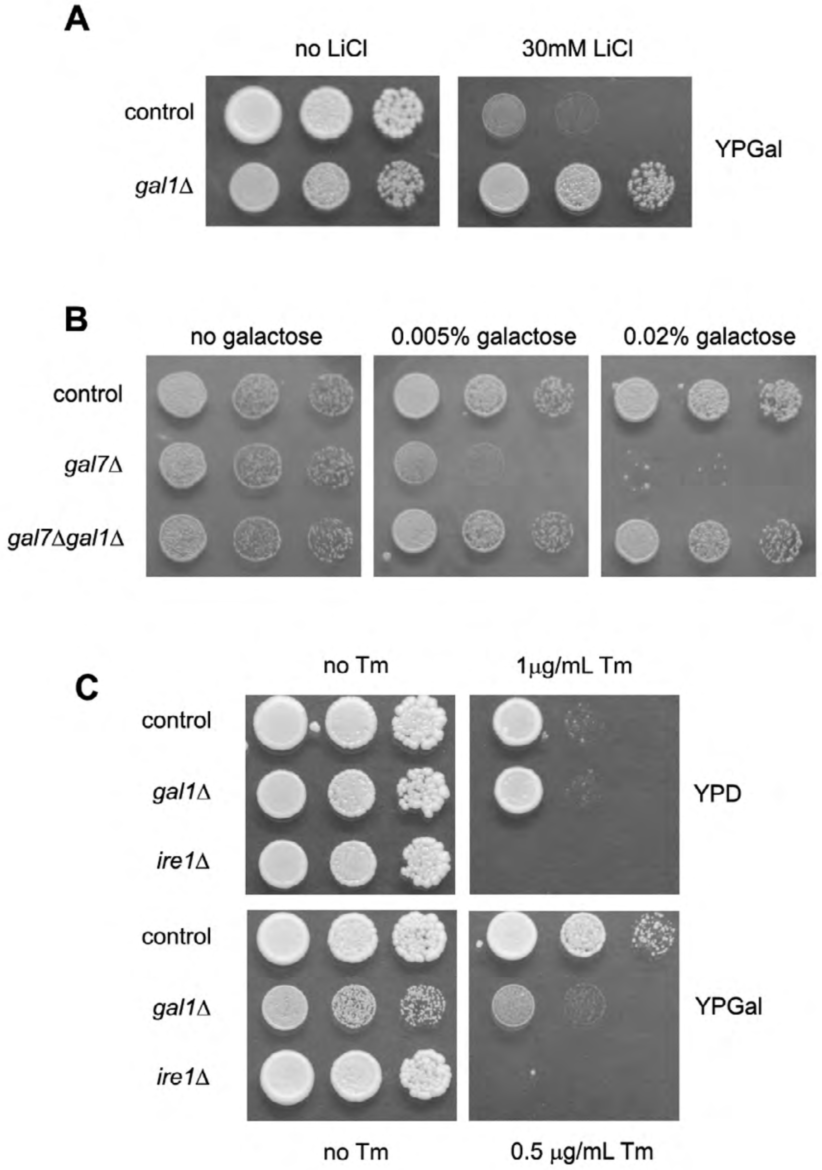

Fig. S1. Deletion of galactokinase suppresses the sensitivity to galactose in both models of galactosemia. (A) Control (*lys2Δ*) and *gal1Δ* yeast strains were grown in YPGal medium until stationary phase and diluted to $O.D._{600nm}$ values of 0.3, 0.03 and 0.003 in sterile water. Approximately 5 μL of each cell suspension were plated in the indicated medium and incubated for 2 (YPD) or 3 (YPGal) days at 30°C. (B) Control, *gal7Δ* and *gal7Δgal1Δ* yeast strains were grown in YPGly medium for 48 hours and diluted to $O.D._{600nm}$ values of 0.3, 0.03 and 0.003 in sterile water. Approximately 5 μL of each cell suspension was plated in YPGly medium plus the indicated amount of galactose and incubated for 4 days at 30°C. (C) Control, *gal1Δ* and *ire1Δ* yeast strains were grown in YPGal medium for 48 hours and diluted to $O.D._{600nm}$ values of 0.3, 0.03 and 0.003 in sterile water. Approximately 5 μL of each cell suspension was plated in YPD or YPGal medium plus the indicated amount of tunicamycin (Tm) and incubated for 3 days at 30°C. Representative results of three independent experiments are shown.

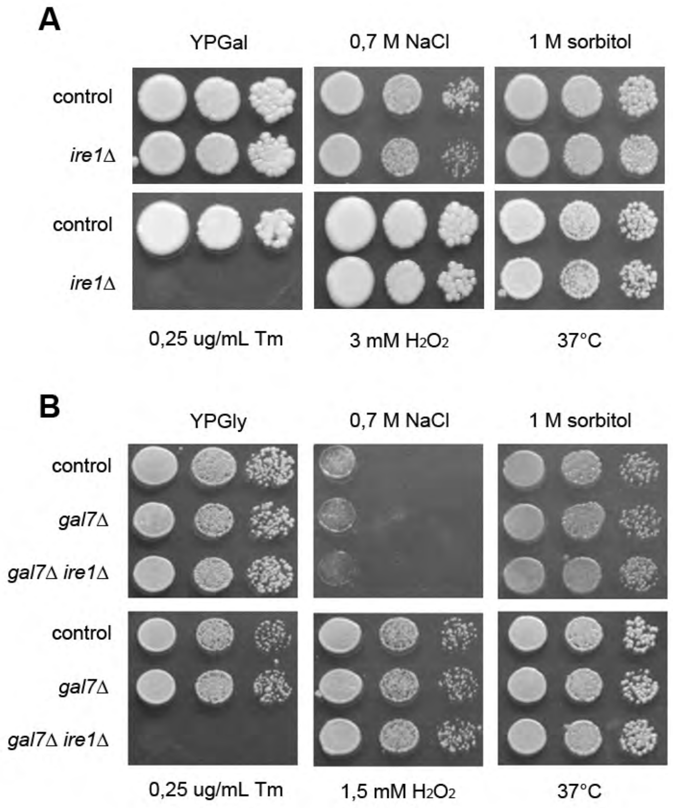

Fig. S2. Deletion of *IRE1* gene does not sensitize yeast cells to high concentrations of NaCl, sorbitol, hydrogen peroxide nor to incubation at 37°C. (A) Control (*lys2Δ*) and *ire1Δ* yeast strains were grown in YPGal medium until stationary phase and diluted to O.D.$_{600nm}$ values of 0.3, 0.03 and 0.003 in sterile water. Approximately 5 µL of each cell suspension were plated in the indicated YPGal based medium and incubated for 3-4 days at 30°C, except for the YPGal plate incubated at 37°C. (B) Control, *gal7Δ* and *gal7Δire1Δ* yeast strains were grown in YPGly medium for 48 hours and diluted to O.D.$_{600nm}$ values of 0.3, 0.03 and 0.003 in sterile water. Approximately 5 µL of each cell suspension was plated in the indicated YPGly based medium and incubated for 4-5 days at 30°C, except for the YPGly plate incubated at 37°C. Representative results of three independent experiments are shown.

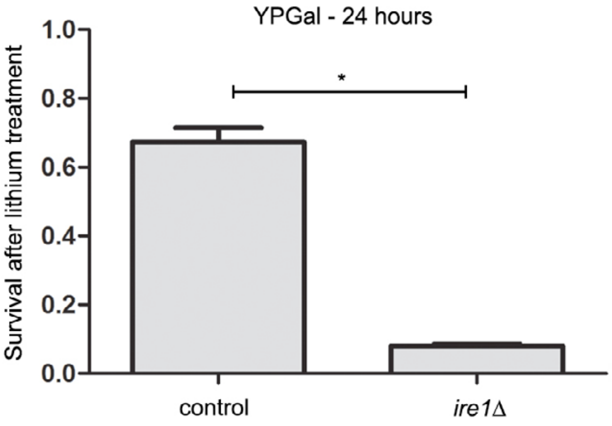

Fig. S3. Impairment of the UPR increases galactose toxicity in the presence of lithium. Control and *ire1Δ* yeast strains were grown in duplicate in YPGal medium until early-log phase (O.D.$_{600nm}$ of 0.1). At this point, LiCl was added to one of the cultures at a final concentration of 30 mM. Aliquots of the cultures were taken 24 hours after the addition of LiCl. Cell suspensions were normalized by O.D.$_{600nm}$ and ~200 cells were inoculated per YPD plate. CFUs were counted after 2 days at 30°C, and the survival rate was calculated by comparing the results of the treated versus untreated conditions. Results are the mean ± s.d. of three independent experiments (* P<0.05, student's *t*-test).

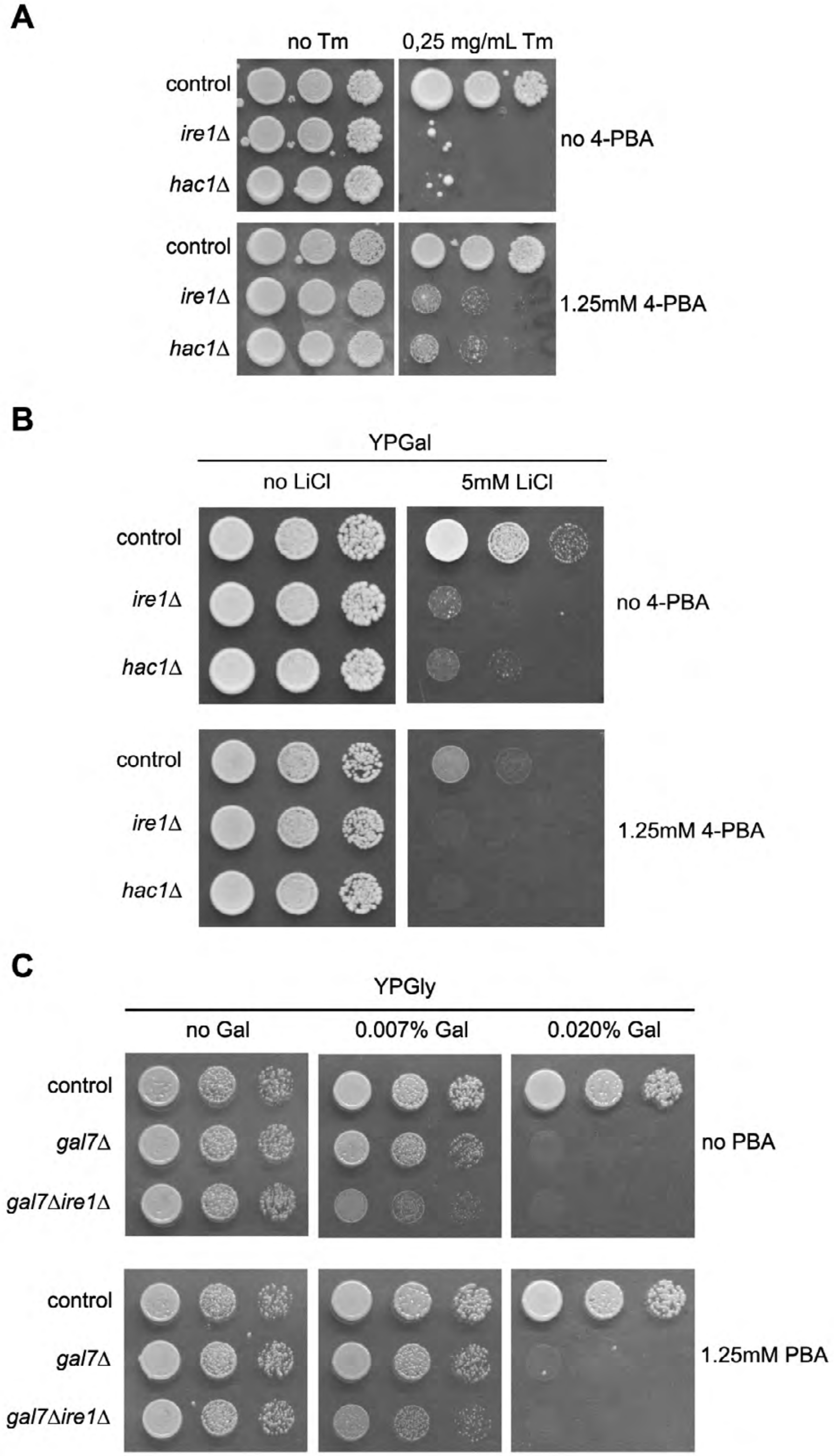

Fig. S4. The chemical chaperone 4-PBA does not suppress galactose toxicity in either model of galactosemia. (A) The indicated yeast strains were grown in YPD medium until stationary phase and diluted to O.D.$_{600nm}$ values of 0.3, 0.03 and 0.003 in sterile water. Approximately 5 μL of each cell suspension was plated in YPD medium supplemented with tunicamycin (Tm) and/or 4-PBA and incubated for 2 days at 30°C. (B) The indicated strains were grown in YPGal medium until stationary phase and diluted to O.D.$_{600nm}$ values of 0.3, 0.03 and 0.003 in sterile water. Approximately 5 μL of each cell suspension was plated in YPGal medium supplemented with LiCl and/or 4-PBA and incubated for 3 days at 30°C. Unexpectedly, 4-PBA and lithium presented a synergistic negative effect on yeast growth. (C) The indicated strains were grown in YPGly medium until stationary phase and diluted to O.D.$_{600nm}$ values of 0.3, 0.03 and 0.003 in sterile water. Approximately 5 μL of each cell suspension was plated in YPGly medium supplemented with galactose and/or 4-PBA and incubated for 4 days at 30°C. Representative results of three independent experiments are shown.

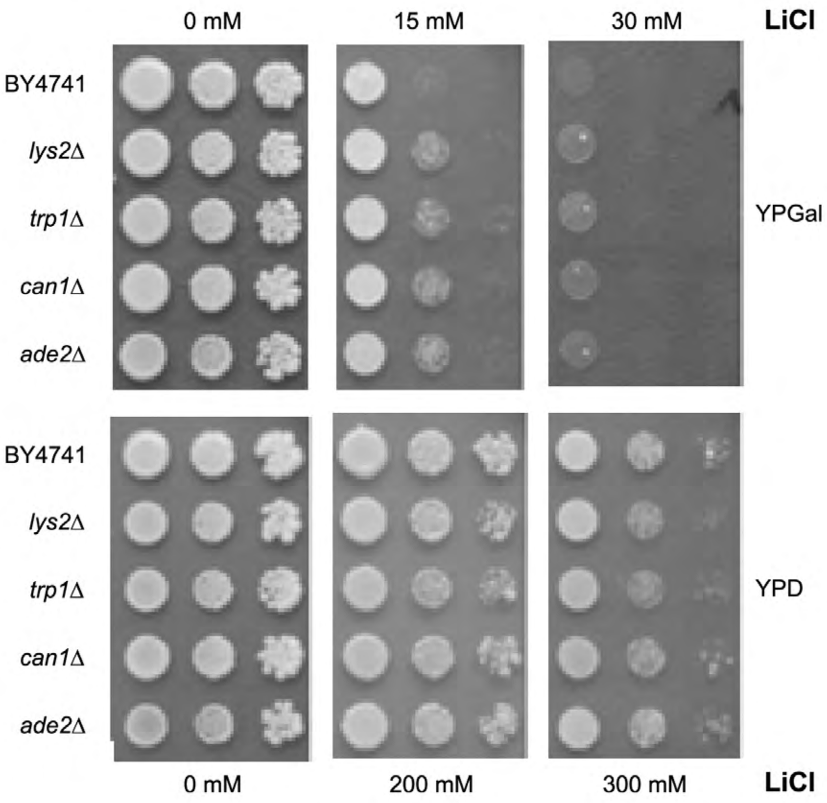

Fig. S5. Most strains from the MatA yeast library are slightly more resistant to LiCl than the parental BY4741 strain. The indicated strains were grown in YPGal medium until stationary phase and diluted to O.D.$_{600nm}$ values of 0.3, 0.03 and 0.003 in sterile water. Approximately 5 µL of each cell suspension was plated in YPD or YPGal medium supplemented with the indicated LiCl concentration and incubated for 2 (YPD) or 3 (YPGal) days at 30°C. We have tested other strains from the library with similar results (data not shown).
